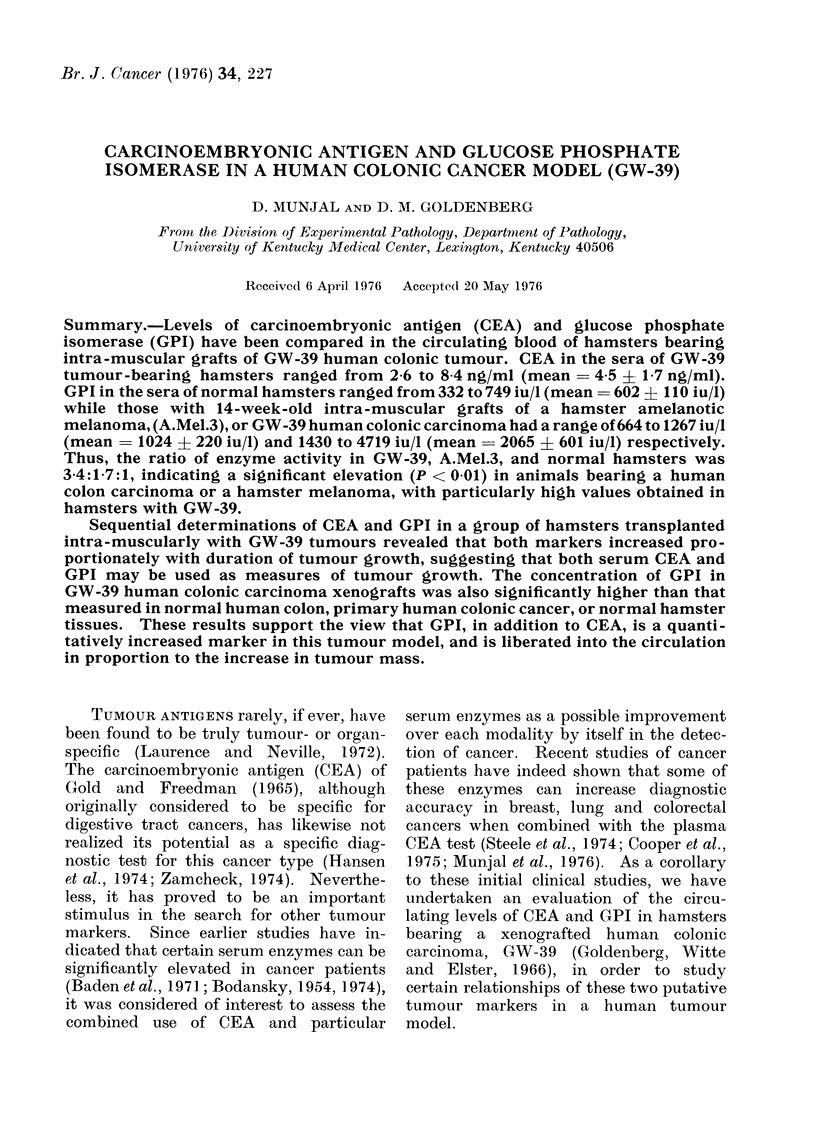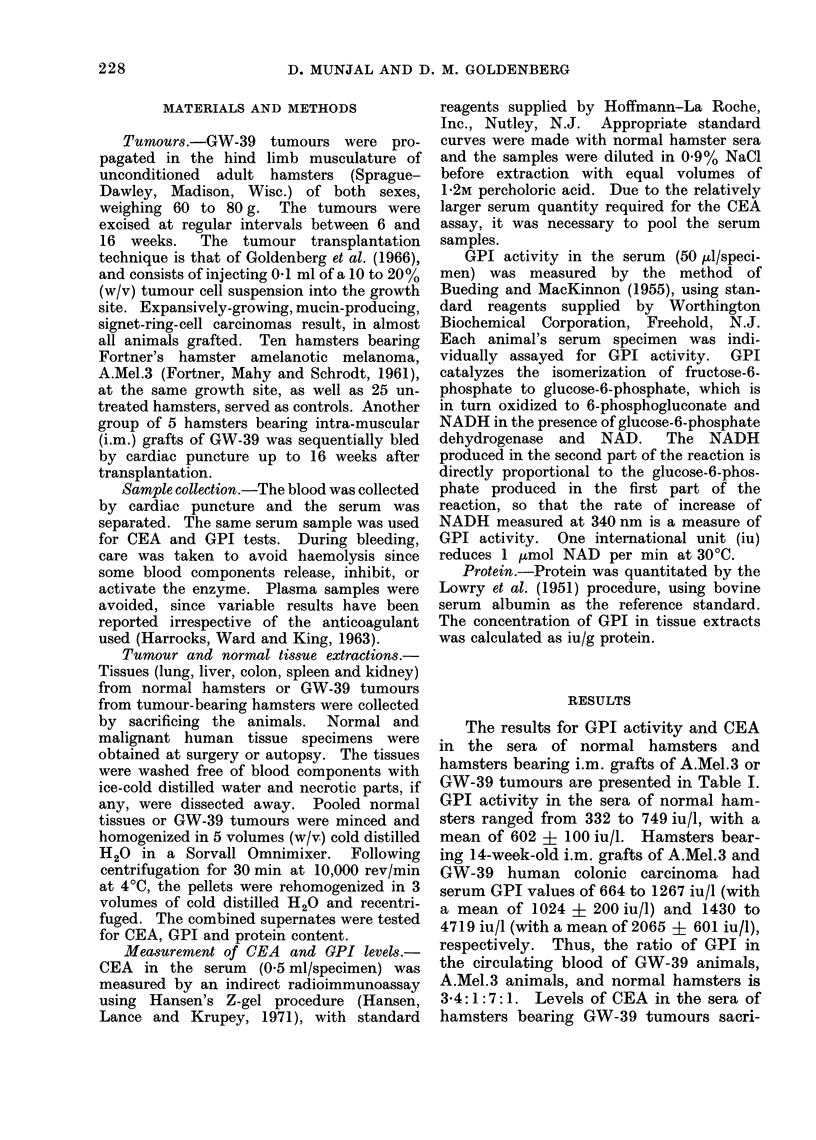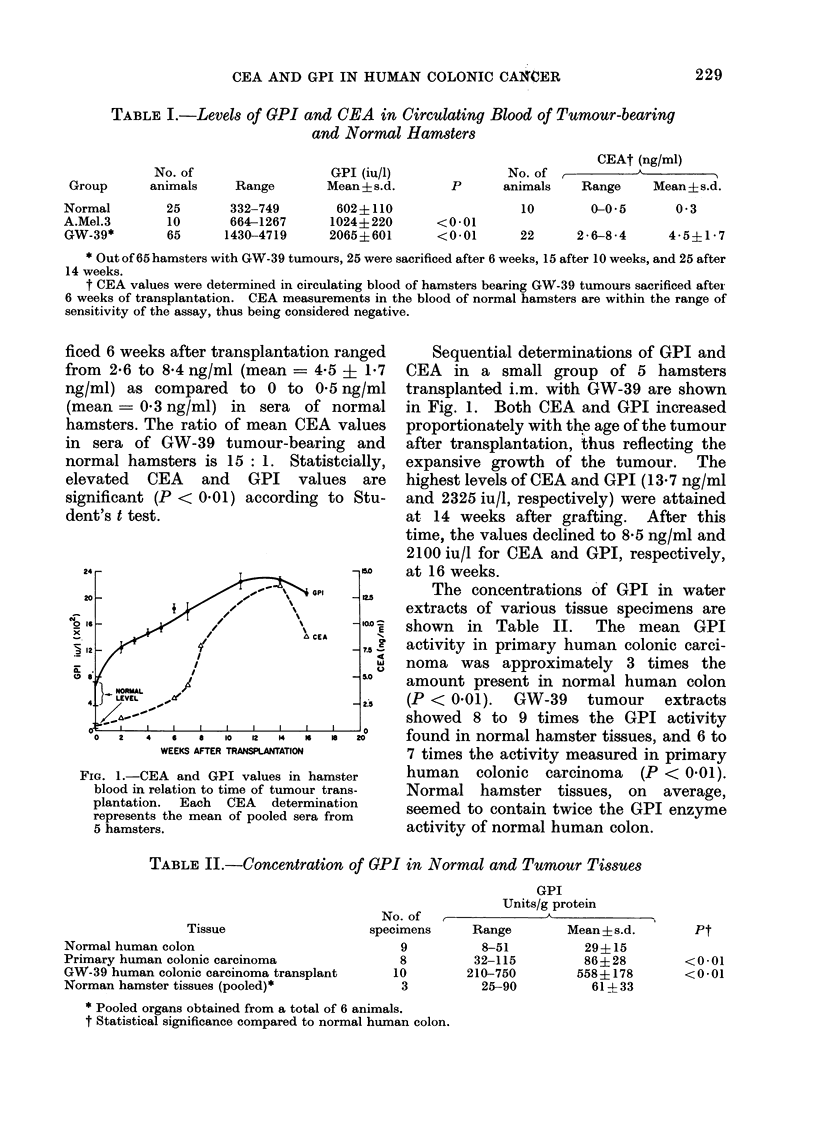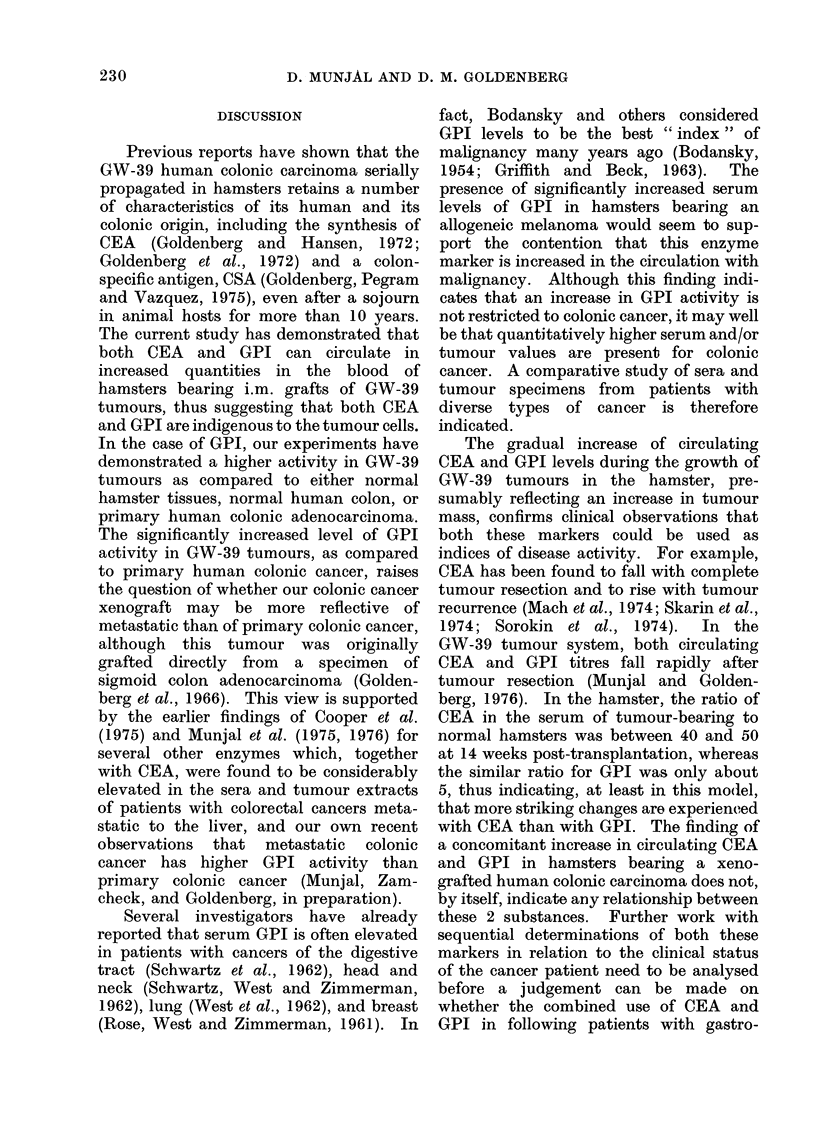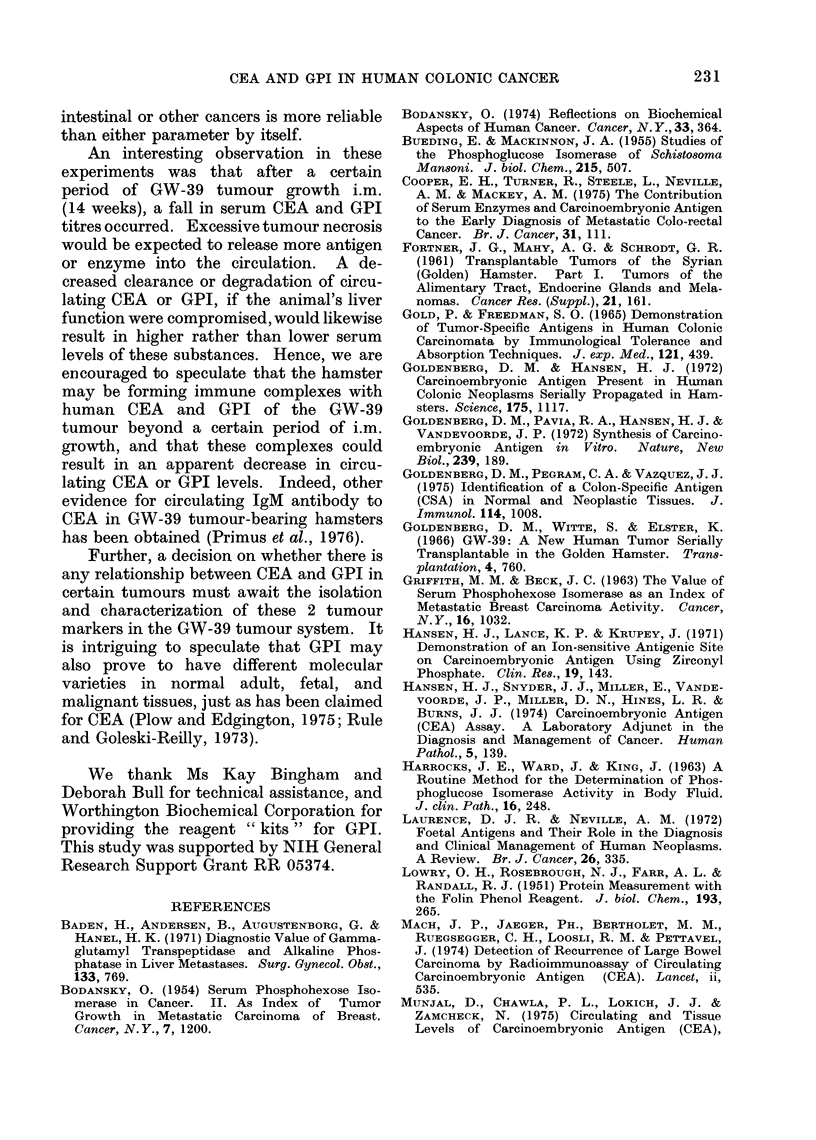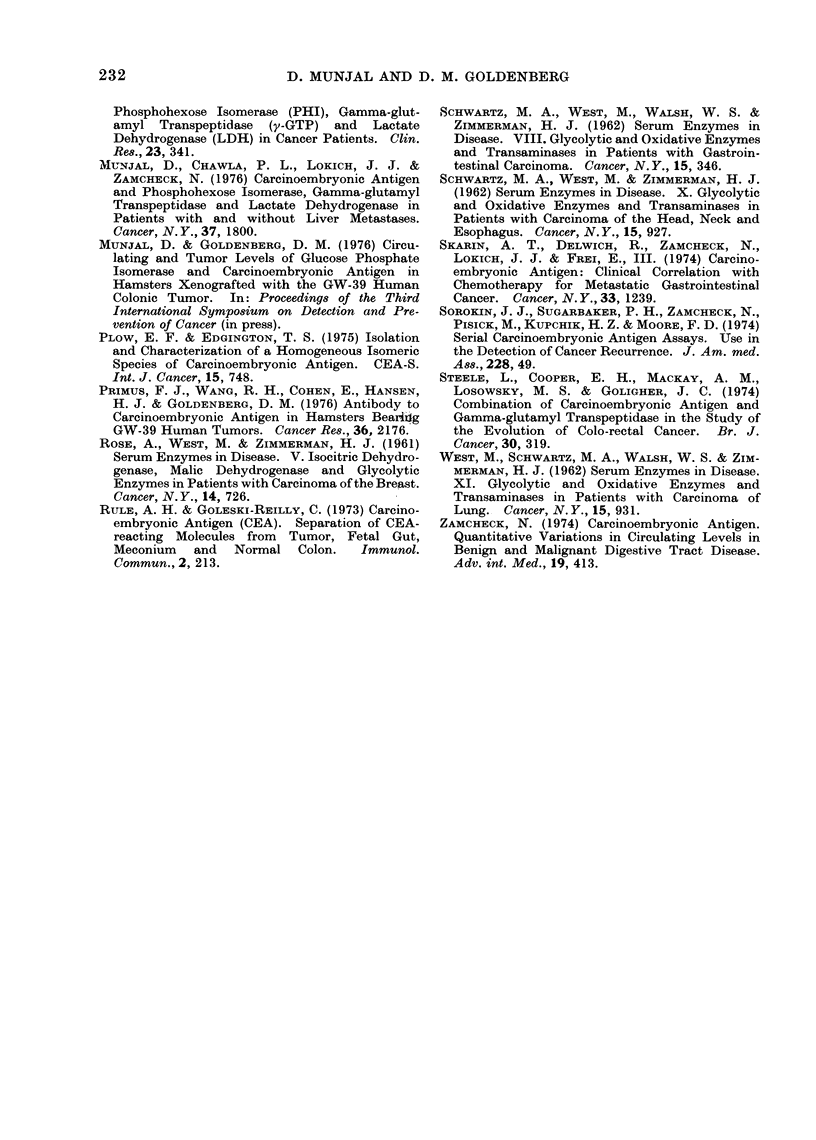# Carcinoembryonic antigen and glucose phosphate isomerase in a human colonic cancer model (GW-39).

**DOI:** 10.1038/bjc.1976.156

**Published:** 1976-09

**Authors:** D. Munjal, D. M. Goldenberg

## Abstract

Levels of carcinoembryonic antigen (CEA) and glucose phosphate isomerase (GPI) have been compared in the circulating blood of hamsters bearing intra-muscular grafts of GW-39 human colonic tumour. CEA in the sera of GW-39 tumour-bearing hamsters ranged from 2-6 to 8-4 ng/ml (mean = 4-5 +/- 1-7 ng/ml). GPI in the sera of normal hamsters ranged from 332 to 749 iu/1 (mean = 602 +/- 110 iu/1) while those with 14-week-old intra-muscular grafts of a hamster amelanotic melanoma, (A.Mel.3), or GW-39 human colonic carcinoma had a range of 664 to 1267 iu/1 (mean = 1024 +/- 220 iu/1) and 1430 to 4719 iu/1 (mean = 2065 +/- 601 iu/1) respectively. Thus, the ratio of enzyme activity in GW-39, A.Mel.3, and normal hamsters was 3-4:1-7:1, indicating a significant elevation (P less than 0-01) in animals bearing a human colon carcinoma or a hamster melanoma, with particularly high values obtained in hamsters with GW-39. Sequential determinations of CEA and GPI in a group of hamsters transplanted intra-muscularly with GW-39 tumours revealed that both markers increased proportionately with duration of tumour growth, suggesting that both serum CEA and GPI may be used as measures of tumour growth. The concentration of GPI in GW-39 human colonic carcinoma xenografts was also significantly higher than that measured in normal human colon, primary human colonic cancer, or normal hamster tissues. These results support the view that GPI, in addition to CEA, is a quantitatively increased marker in this tumour model, and is liberated into the circulation in proportion to the increase in tumour mass.


Br. J. Cancer (1976) 34, 227

### CARCINOEMBRYONIC ANTIGEN AND GLUCOSE PHOSPHATE ISOMERASE IN A HUMAN COLONIC CANCER MODEL (GW-39)

D. MUNJAL AND D. M. GOLDENBERG

*From the Division of Experimental Pathology, Department of Pathology, University of Kentucky Medical Center, Lexington, Kentucky* 40506

Received 6 April 1976 Accepted 20 May 1976

**Summary.—Levels of carcinoembryonic antigen (CEA) and glucose phosphate isomerase (GPI) have been compared in the circulating blood of hamsters bearing intra-muscular grafts of GW-39 human colonic tumour. CEA in the sera of GW-39 tumour-bearing hamsters ranged from 2·6 to 8·4 ng/ml (mean = 4·5 ± 1·7 ng/ml). GPI in the sera of normal hamsters ranged from 332 to 749 iu/l (mean = 602 ± 110 iu/l) while those with 14-week-old intra-muscular grafts of a hamster amelanotic melanoma, (A.Mel.3), or GW-39 human colonic carcinoma had a range of 664 to 1267 iu/l (mean = 1024 ± 220 iu/l) and 1430 to 4719 iu/l (mean = 2065 ± 601 iu/l) respectively. Thus, the ratio of enzyme activity in GW-39, A.Mel.3, and normal hamsters was 3·4:1·7:1, indicating a significant elevation ($P < 0{\cdot}01$) in animals bearing a human colon carcinoma or a hamster melanoma, with particularly high values obtained in hamsters with GW-39.**

**Sequential determinations of CEA and GPI in a group of hamsters transplanted intra-muscularly with GW-39 tumours revealed that both markers increased proportionately with duration of tumour growth, suggesting that both serum CEA and GPI may be used as measures of tumour growth. The concentration of GPI in GW-39 human colonic carcinoma xenografts was also significantly higher than that measured in normal human colon, primary human colonic cancer, or normal hamster tissues. These results support the view that GPI, in addition to CEA, is a quantitatively increased marker in this tumour model, and is liberated into the circulation in proportion to the increase in tumour mass.**

TUMOUR ANTIGENS rarely, if ever, have been found to be truly tumour- or organ-specific (Laurence and Neville, 1972). The carcinoembryonic antigen (CEA) of Gold and Freedman (1965), although originally considered to be specific for digestive tract cancers, has likewise not realized its potential as a specific diagnostic test for this cancer type (Hansen *et al.*, 1974; Zamcheck, 1974). Nevertheless, it has proved to be an important stimulus in the search for other tumour markers. Since earlier studies have indicated that certain serum enzymes can be significantly elevated in cancer patients (Baden *et al.*, 1971; Bodansky, 1954, 1974), it was considered of interest to assess the combined use of CEA and particular serum enzymes as a possible improvement over each modality by itself in the detection of cancer. Recent studies of cancer patients have indeed shown that some of these enzymes can increase diagnostic accuracy in breast, lung and colorectal cancers when combined with the plasma CEA test (Steele *et al.*, 1974; Cooper *et al.*, 1975; Munjal *et al.*, 1976). As a corollary to these initial clinical studies, we have undertaken an evaluation of the circulating levels of CEA and GPI in hamsters bearing a xenografted human colonic carcinoma, GW-39 (Goldenberg, Witte and Elster, 1966), in order to study certain relationships of these two putative tumour markers in a human tumour model.

### MATERIALS AND METHODS

*Tumours*.—GW-39 tumours were propagated in the hind limb musculature of unconditioned adult hamsters (Sprague–Dawley, Madison, Wisc.) of both sexes, weighing 60 to 80 g. The tumours were excised at regular intervals between 6 and 16 weeks. The tumour transplantation technique is that of Goldenberg *et al.* (1966), and consists of injecting 0·1 ml of a 10 to 20% (w/v) tumour cell suspension into the growth site. Expansively-growing, mucin-producing, signet-ring-cell carcinomas result, in almost all animals grafted. Ten hamsters bearing Fortner's hamster amelanotic melanoma, A.Mel.3 (Fortner, Mahy and Schrodt, 1961), at the same growth site, as well as 25 untreated hamsters, served as controls. Another group of 5 hamsters bearing intra-muscular (i.m.) grafts of GW-39 was sequentially bled by cardiac puncture up to 16 weeks after transplantation.

*Sample collection*.—The blood was collected by cardiac puncture and the serum was separated. The same serum sample was used for CEA and GPI tests. During bleeding, care was taken to avoid haemolysis since some blood components release, inhibit, or activate the enzyme. Plasma samples were avoided, since variable results have been reported irrespective of the anticoagulant used (Harrocks, Ward and King, 1963).

*Tumour and normal tissue extractions*.—Tissues (lung, liver, colon, spleen and kidney) from normal hamsters or GW-39 tumours from tumour-bearing hamsters were collected by sacrificing the animals. Normal and malignant human tissue specimens were obtained at surgery or autopsy. The tissues were washed free of blood components with ice-cold distilled water and necrotic parts, if any, were dissected away. Pooled normal tissues or GW-39 tumours were minced and homogenized in 5 volumes (w/v) cold distilled $H_2O$ in a Sorvall Omnimixer. Following centrifugation for 30 min at 10,000 rev/min at 4°C, the pellets were rehomogenized in 3 volumes of cold distilled $H_2O$ and recentrifuged. The combined supernates were tested for CEA, GPI and protein content.

*Measurement of CEA and GPI levels*.—CEA in the serum (0·5 ml/specimen) was measured by an indirect radioimmunoassay using Hansen's Z-gel procedure (Hansen, Lance and Krupey, 1971), with standard reagents supplied by Hoffmann–La Roche, Inc., Nutley, N.J. Appropriate standard curves were made with normal hamster sera and the samples were diluted in 0·9% NaCl before extraction with equal volumes of 1·2M percholoric acid. Due to the relatively larger serum quantity required for the CEA assay, it was necessary to pool the serum samples.

GPI activity in the serum (50 μl/specimen) was measured by the method of Bueding and MacKinnon (1955), using standard reagents supplied by Worthington Biochemical Corporation, Freehold, N.J. Each animal's serum specimen was individually assayed for GPI activity. GPI catalyzes the isomerization of fructose-6-phosphate to glucose-6-phosphate, which is in turn oxidized to 6-phosphogluconate and NADH in the presence of glucose-6-phosphate dehydrogenase and NAD. The NADH produced in the second part of the reaction is directly proportional to the glucose-6-phosphate produced in the first part of the reaction, so that the rate of increase of NADH measured at 340 nm is a measure of GPI activity. One international unit (iu) reduces 1 μmol NAD per min at 30°C.

*Protein*.—Protein was quantitated by the Lowry *et al.* (1951) procedure, using bovine serum albumin as the reference standard. The concentration of GPI in tissue extracts was calculated as iu/g protein.

### RESULTS

The results for GPI activity and CEA in the sera of normal hamsters and hamsters bearing i.m. grafts of A.Mel.3 or GW-39 tumours are presented in Table I. GPI activity in the sera of normal hamsters ranged from 332 to 749 iu/l, with a mean of 602 ± 100 iu/l. Hamsters bearing 14-week-old i.m. grafts of A.Mel.3 and GW-39 human colonic carcinoma had serum GPI values of 664 to 1267 iu/l (with a mean of 1024 ± 200 iu/l) and 1430 to 4719 iu/l (with a mean of 2065 ± 601 iu/l), respectively. Thus, the ratio of GPI in the circulating blood of GW-39 animals, A.Mel.3 animals, and normal hamsters is 3·4:1:7:1. Levels of CEA in the sera of hamsters bearing GW-39 tumours sacri-

TABLE I.—*Levels of GPI and CEA in Circulating Blood of Tumour-bearing and Normal Hamsters*

| Group | No. of animals | Range | GPI (iu/l) Mean ± s.d. | *P* | No. of animals | CEA† (ng/ml) Range | CEA† (ng/ml) Mean ± s.d. |
|---|---|---|---|---|---|---|---|
| Normal | 25 | 332–749 | 602±110 | | 10 | 0–0·5 | 0·3 |
| A.Mel.3 | 10 | 664–1267 | 1024±220 | <0·01 | | | |
| GW-39* | 65 | 1430–4719 | 2065±601 | <0·01 | 22 | 2·6–8·4 | 4·5±1·7 |

* Out of 65 hamsters with GW-39 tumours, 25 were sacrificed after 6 weeks, 15 after 10 weeks, and 25 after 14 weeks.

† CEA values were determined in circulating blood of hamsters bearing GW-39 tumours sacrificed after 6 weeks of transplantation. CEA measurements in the blood of normal hamsters are within the range of sensitivity of the assay, thus being considered negative.

ficed 6 weeks after transplantation ranged from 2·6 to 8·4 ng/ml (mean = 4·5 ± 1·7 ng/ml) as compared to 0 to 0·5 ng/ml (mean = 0·3 ng/ml) in sera of normal hamsters. The ratio of mean CEA values in sera of GW-39 tumour-bearing and normal hamsters is 15 : 1. Statistcially, elevated CEA and GPI values are significant ($P < 0{\cdot}01$) according to Student's *t* test.

FIG. 1.—CEA and GPI values in hamster blood in relation to time of tumour transplantation. Each CEA determination represents the mean of pooled sera from 5 hamsters.

Sequential determinations of GPI and CEA in a small group of 5 hamsters transplanted i.m. with GW-39 are shown in Fig. 1. Both CEA and GPI increased proportionately with the age of the tumour after transplantation, thus reflecting the expansive growth of the tumour. The highest levels of CEA and GPI (13·7 ng/ml and 2325 iu/l, respectively) were attained at 14 weeks after grafting. After this time, the values declined to 8·5 ng/ml and 2100 iu/l for CEA and GPI, respectively, at 16 weeks.

The concentrations of GPI in water extracts of various tissue specimens are shown in Table II. The mean GPI activity in primary human colonic carcinoma was approximately 3 times the amount present in normal human colon ($P < 0{\cdot}01$). GW-39 tumour extracts showed 8 to 9 times the GPI activity found in normal hamster tissues, and 6 to 7 times the activity measured in primary human colonic carcinoma ($P < 0{\cdot}01$). Normal hamster tissues, on average, seemed to contain twice the GPI enzyme activity of normal human colon.

TABLE II.—*Concentration of GPI in Normal and Tumour Tissues*

| Tissue | No. of specimens | GPI Units/g protein Range | GPI Units/g protein Mean ± s.d. | *P*† |
|---|---|---|---|---|
| Normal human colon | 9 | 8–51 | 29±15 | |
| Primary human colonic carcinoma | 8 | 32–115 | 86±28 | <0·01 |
| GW-39 human colonic carcinoma transplant | 10 | 210–750 | 558±178 | <0·01 |
| Norman hamster tissues (pooled)* | 3 | 25–90 | 61±33 | |

* Pooled organs obtained from a total of 6 animals.

† Statistical significance compared to normal human colon.

### DISCUSSION

Previous reports have shown that the GW-39 human colonic carcinoma serially propagated in hamsters retains a number of characteristics of its human and its colonic origin, including the synthesis of CEA (Goldenberg and Hansen, 1972; Goldenberg *et al.*, 1972) and a colon-specific antigen, CSA (Goldenberg, Pegram and Vazquez, 1975), even after a sojourn in animal hosts for more than 10 years. The current study has demonstrated that both CEA and GPI can circulate in increased quantities in the blood of hamsters bearing i.m. grafts of GW-39 tumours, thus suggesting that both CEA and GPI are indigenous to the tumour cells. In the case of GPI, our experiments have demonstrated a higher activity in GW-39 tumours as compared to either normal hamster tissues, normal human colon, or primary human colonic adenocarcinoma. The significantly increased level of GPI activity in GW-39 tumours, as compared to primary human colonic cancer, raises the question of whether our colonic cancer xenograft may be more reflective of metastatic than of primary colonic cancer, although this tumour was originally grafted directly from a specimen of sigmoid colon adenocarcinoma (Goldenberg *et al.*, 1966). This view is supported by the earlier findings of Cooper *et al.* (1975) and Munjal *et al.* (1975, 1976) for several other enzymes which, together with CEA, were found to be considerably elevated in the sera and tumour extracts of patients with colorectal cancers metastatic to the liver, and our own recent observations that metastatic colonic cancer has higher GPI activity than primary colonic cancer (Munjal, Zamcheck, and Goldenberg, in preparation).

Several investigators have already reported that serum GPI is often elevated in patients with cancers of the digestive tract (Schwartz *et al.*, 1962), head and neck (Schwartz, West and Zimmerman, 1962), lung (West *et al.*, 1962), and breast (Rose, West and Zimmerman, 1961). In fact, Bodansky and others considered GPI levels to be the best " index " of malignancy many years ago (Bodansky, 1954; Griffith and Beck, 1963). The presence of significantly increased serum levels of GPI in hamsters bearing an allogeneic melanoma would seem to support the contention that this enzyme marker is increased in the circulation with malignancy. Although this finding indicates that an increase in GPI activity is not restricted to colonic cancer, it may well be that quantitatively higher serum and/or tumour values are present for colonic cancer. A comparative study of sera and tumour specimens from patients with diverse types of cancer is therefore indicated.

The gradual increase of circulating CEA and GPI levels during the growth of GW-39 tumours in the hamster, presumably reflecting an increase in tumour mass, confirms clinical observations that both these markers could be used as indices of disease activity. For example, CEA has been found to fall with complete tumour resection and to rise with tumour recurrence (Mach *et al.*, 1974; Skarin *et al.*, 1974; Sorokin *et al.*, 1974). In the GW-39 tumour system, both circulating CEA and GPI titres fall rapidly after tumour resection (Munjal and Goldenberg, 1976). In the hamster, the ratio of CEA in the serum of tumour-bearing to normal hamsters was between 40 and 50 at 14 weeks post-transplantation, whereas the similar ratio for GPI was only about 5, thus indicating, at least in this model, that more striking changes are experienced with CEA than with GPI. The finding of a concomitant increase in circulating CEA and GPI in hamsters bearing a xenografted human colonic carcinoma does not, by itself, indicate any relationship between these 2 substances. Further work with sequential determinations of both these markers in relation to the clinical status of the cancer patient need to be analysed before a judgement can be made on whether the combined use of CEA and GPI in following patients with gastrointestinal or other cancers is more reliable than either parameter by itself.

An interesting observation in these experiments was that after a certain period of GW-39 tumour growth i.m. (14 weeks), a fall in serum CEA and GPI titres occurred. Excessive tumour necrosis would be expected to release more antigen or enzyme into the circulation. A decreased clearance or degradation of circulating CEA or GPI, if the animal's liver function were compromised, would likewise result in higher rather than lower serum levels of these substances. Hence, we are encouraged to speculate that the hamster may be forming immune complexes with human CEA and GPI of the GW-39 tumour beyond a certain period of i.m. growth, and that these complexes could result in an apparent decrease in circulating CEA or GPI levels. Indeed, other evidence for circulating IgM antibody to CEA in GW-39 tumour-bearing hamsters has been obtained (Primus *et al.*, 1976).

Further, a decision on whether there is any relationship between CEA and GPI in certain tumours must await the isolation and characterization of these 2 tumour markers in the GW-39 tumour system. It is intriguing to speculate that GPI may also prove to have different molecular varieties in normal adult, fetal, and malignant tissues, just as has been claimed for CEA (Plow and Edgington, 1975; Rule and Goleski-Reilly, 1973).

We thank Ms Kay Bingham and Deborah Bull for technical assistance, and Worthington Biochemical Corporation for providing the reagent " kits " for GPI. This study was supported by NIH General Research Support Grant RR 05374.

REFERENCES

BADEN, H., ANDERSEN, B., AUGUSTENBORG, G. & HANEL, H. K. (1971) Diagnostic Value of Gamma-glutamyl Transpeptidase and Alkaline Phosphatase in Liver Metastases. *Surg. Gynecol. Obst.*, **133**, 769.

BODANSKY, O. (1954) Serum Phosphohexose Isomerase in Cancer. II. As Index of Tumor Growth in Metastatic Carcinoma of Breast. *Cancer, N.Y.*, **7**, 1200.

BODANSKY, O. (1974) Reflections on Biochemical Aspects of Human Cancer. *Cancer, N.Y.*, **33**, 364.

BUEDING, E. & MACKINNON, J. A. (1955) Studies of the Phosphoglucose Isomerase of *Schistosoma Mansoni*. *J. biol. Chem.*, **215**, 507.

COOPER, E. H., TURNER, R., STEELE, L., NEVILLE, A. M. & MACKEY, A. M. (1975) The Contribution of Serum Enzymes and Carcinoembryonic Antigen to the Early Diagnosis of Metastatic Colo-rectal Cancer. *Br. J. Cancer*, **31**, 111.

FORTNER, J. G., MAHY, A. G. & SCHRODT, G. R. (1961) Transplantable Tumors of the Syrian (Golden) Hamster. Part I. Tumors of the Alimentary Tract, Endocrine Glands and Melanomas. *Cancer Res.* (*Suppl.*), **21**, 161.

GOLD, P. & FREEDMAN, S. O. (1965) Demonstration of Tumor-Specific Antigens in Human Colonic Carcinomata by Immunological Tolerance and Absorption Techniques. *J. exp. Med.*, **121**, 439.

GOLDENBERG, D. M. & HANSEN, H. J. (1972) Carcinoembryonic Antigen Present in Human Colonic Neoplasms Serially Propagated in Hamsters. *Science*, **175**, 1117.

GOLDENBERG, D. M., PAVIA, R. A., HANSEN, H. J. & VANDEVOORDE, J. P. (1972) Synthesis of Carcinoembryonic Antigen *in Vitro*. *Nature, New Biol.*, **239**, 189.

GOLDENBERG, D. M., PEGRAM, C. A. & VAZQUEZ, J. J. (1975) Identification of a Colon-Specific Antigen (CSA) in Normal and Neoplastic Tissues. *J. Immunol.* **114**, 1008.

GOLDENBERG, D. M., WITTE, S. & ELSTER, K. (1966) GW-39: A New Human Tumor Serially Transplantable in the Golden Hamster. *Transplantation*, **4**, 760.

GRIFFITH, M. M. & BECK, J. C. (1963) The Value of Serum Phosphohexose Isomerase as an Index of Metastatic Breast Carcinoma Activity. *Cancer, N.Y.*, **16**, 1032.

HANSEN, H. J., LANCE, K. P. & KRUPEY, J. (1971) Demonstration of an Ion-sensitive Antigenic Site on Carcinoembryonic Antigen Using Zirconyl Phosphate. *Clin. Res.*, **19**, 143.

HANSEN, H. J., SNYDER, J. J., MILLER, E., VANDEVOORDE, J. P., MILLER, D. N., HINES, L. R. & BURNS, J. J. (1974) Carcinoembryonic Antigen (CEA) Assay. A Laboratory Adjunct in the Diagnosis and Management of Cancer. *Human Pathol.*, **5**, 139.

HARROCKS, J. E., WARD, J. & KING, J. (1963) A Routine Method for the Determination of Phosphoglucose Isomerase Activity in Body Fluid. *J. clin. Path.*, **16**, 248.

LAURENCE, D. J. R. & NEVILLE, A. M. (1972) Foetal Antigens and Their Role in the Diagnosis and Clinical Management of Human Neoplasms. A Review. *Br. J. Cancer*, **26**, 335.

LOWRY, O. H., ROSEBROUGH, N. J., FARR, A. L. & RANDALL, R. J. (1951) Protein Measurement with the Folin Phenol Reagent. *J. biol. Chem.*, **193**, 265.

MACH, J. P., JAEGER, PH., BERTHOLET, M. M., RUEGSEGGER, C. H., LOOSLI, R. M. & PETTAVEL, J. (1974) Detection of Recurrence of Large Bowel Carcinoma by Radioimmunoassay of Circulating Carcinoembryonic Antigen (CEA). *Lancet*, ii, 535.

MUNJAL, D., CHAWLA, P. L., LOKICH, J. J. & ZAMCHECK, N. (1975) Circulating and Tissue Levels of Carcinoembryonic Antigen (CEA),

Phosphohexose Isomerase (PHI), Gamma-glutamyl Transpeptidase ($\gamma$-GTP) and Lactate Dehydrogenase (LDH) in Cancer Patients. *Clin. Res.*, **23**, 341.

MUNJAL, D., CHAWLA, P. L., LOKICH, J. J. & ZAMCHECK, N. (1976) Carcinoembryonic Antigen and Phosphohexose Isomerase, Gamma-glutamyl Transpeptidase and Lactate Dehydrogenase in Patients with and without Liver Metastases. *Cancer, N.Y.*, **37**, 1800.

MUNJAL, D. & GOLDENBERG, D. M. (1976) Circulating and Tumor Levels of Glucose Phosphate Isomerase and Carcinoembryonic Antigen in Hamsters Xenografted with the GW-39 Human Colonic Tumor. In: *Proceedings of the Third International Symposium on Detection and Prevention of Cancer* (in press).

PLOW, E. F. & EDGINGTON, T. S. (1975) Isolation and Characterization of a Homogeneous Isomeric Species of Carcinoembryonic Antigen. CEA-S. *Int. J. Cancer*, **15**, 748.

PRIMUS, F. J., WANG, R. H., COHEN, E., HANSEN, H. J. & GOLDENBERG, D. M. (1976) Antibody to Carcinoembryonic Antigen in Hamsters Bearing GW-39 Human Tumors. *Cancer Res.*, **36**, 2176.

ROSE, A., WEST, M. & ZIMMERMAN, H. J. (1961) Serum Enzymes in Disease. V. Isocitric Dehydrogenase, Malic Dehydrogenase and Glycolytic Enzymes in Patients with Carcinoma of the Breast. *Cancer, N.Y.*, **14**, 726.

RULE, A. H. & GOLESKI-REILLY, C. (1973) Carcinoembryonic Antigen (CEA). Separation of CEA-reacting Molecules from Tumor, Fetal Gut, Meconium and Normal Colon. *Immunol. Commun.*, **2**, 213.

SCHWARTZ, M. A., WEST, M., WALSH, W. S. & ZIMMERMAN, H. J. (1962) Serum Enzymes in Disease. VIII. Glycolytic and Oxidative Enzymes and Transaminases in Patients with Gastrointestinal Carcinoma. *Cancer, N.Y.*, **15**, 346.

SCHWARTZ, M. A., WEST, M. & ZIMMERMAN, H. J. (1962) Serum Enzymes in Disease. X. Glycolytic and Oxidative Enzymes and Transaminases in Patients with Carcinoma of the Head, Neck and Esophagus. *Cancer, N.Y.*, **15**, 927.

SKARIN, A. T., DELWICH, R., ZAMCHECK, N., LOKICH, J. J. & FREI, E., III. (1974) Carcinoembryonic Antigen: Clinical Correlation with Chemotherapy for Metastatic Gastrointestinal Cancer. *Cancer, N.Y.*, **33**, 1239.

SOROKIN, J. J., SUGARBAKER, P. H., ZAMCHECK, N., PISICK, M., KUPCHIK, H. Z. & MOORE, F. D. (1974) Serial Carcinoembryonic Antigen Assays. Use in the Detection of Cancer Recurrence. *J. Am. med. Ass.*, **228**, 49.

STEELE, L., COOPER, E. H., MACKAY, A. M., LOSOWSKY, M. S. & GOLIGHER, J. C. (1974) Combination of Carcinoembryonic Antigen and Gamma-glutamyl Transpeptidase in the Study of the Evolution of Colo-rectal Cancer. *Br. J. Cancer*, **30**, 319.

WEST, M., SCHWARTZ, M. A., WALSH, W. S. & ZIMMERMAN, H. J. (1962) Serum Enzymes in Disease. XI. Glycolytic and Oxidative Enzymes and Transaminases in Patients with Carcinoma of Lung. *Cancer, N.Y.*, **15**, 931.

ZAMCHECK, N. (1974) Carcinoembryonic Antigen. Quantitative Variations in Circulating Levels in Benign and Malignant Digestive Tract Disease. *Adv. int. Med.*, **19**, 413.